# Heat Shock Protein Chaperome Is a Multi-Faceted Vector for Tumor Cell Migratory Activity, Invasion, and Metastasis

**DOI:** 10.3390/cells14231837

**Published:** 2025-11-21

**Authors:** Viacheslav Fedorov, Andrey Kurkin, Georgii Fofanov, Vitaliya Kaneva, Anna Kondratenko, Stephanie E. Combs, Maxim Shevtsov

**Affiliations:** 1 Department of Inorganic Chemistry and Biophysics, Saint-Petersburg State University of Veterinary Medicine, 196084 Saint-Petersburg, Russia; v.fedorov@spbguvm.ru; 2 Laboratory of Biomedical Nanotechnologies, Institute of Cytology of the Russian Academy of Sciences (RAS), 194064 Saint-Petersburg, Russia; andrey-k0@mail.ru; 3 Personalized Medicine Centre, Almazov National Medical Research Centre, 197341 Saint-Petersburg, Russia; fofanovgeorgij@gmail.com; 4 Saint-Petersburg State University, 199034 Saint-Petersburg, Russia; 5 Molecular Biotechnology Department, Saint-Petersburg State Technological Institute (Technical University), 190013 Saint-Petersburg, Russia; kaneva_vd@mail.ru (V.K.); anna_kondratenko_03@mail.ru (A.K.); 6 Department of Radiation Oncology, Technische Universität München (TUM), Klinikum Rechts der Isar, 81675 Munich, Germany; stephanie.combs@tum.de

**Keywords:** heat shock proteins, chaperome, HSP70, HSP90, molecular network, cancer cell migration, invasion, cancer progression

## Abstract

Heat shock proteins (HSPs), in particular, representatives of the HSP70 and HSP90 families, are the folding centers of cell proteins and have been proven to be overexpressed in various types of solid and hematological malignancies. With their involvement in a number of cellular functions (e.g., protection from various stresses including radiochemotherapy, transport regulation, apoptotic signal inhibition, etc.), these chaperones are a valuable target for cancer progression research. However, recent focus has shifted to the HSP interaction network, which includes many molecules involved in cell migration and invasion pathways. Investigating the interplay between different co-chaperones and their effect on cell motility may help with establishing a palette of available diagnostic and therapeutic targets for highly invasive cancer types. In this review, we describe current models of the HSP functional cycle and recent studies proving links between these cycle regulators and contributions to cell migration. Based on detailed studies of various co-chaperones’ involvement in cancer progression, the network approach gives much necessary molecular context to previously established HSP functions.

## 1. Introduction

Cancer is a major cause of mortality in many countries and is characterized by abnormal cell growth with significant variations in incidence and mortality rates across different demographics and geographic regions. In recent decades, statistics show a stabilizing incidence of solid tumors in the EU and US (approx. 300 cases per 100,000 patients) with declining mortality rates, yet cancer deaths remain the second most common after heart diseases [1]. Common cancers include lung, colorectal, breast, melanoma, and prostate cancers, with incidence rates escalating with age and substantial disparities observed based on sex and ethnicity [2]. Epidemiological studies utilize cancer registries to track incidence and mortality, revealing that smoking remains the leading modifiable risk factor associated with nearly half of all cancer deaths, while metabolic and cell division dysregulations are listed as main endogenous cancer causes. Prevention strategies focus on reducing the exposure to risk factors, implementing screening programs, and promoting vaccinations to combat infections linked to cancer. Effective detection and treatment strategies are vital for improving cancer outcomes. Working with the diagnosis and consequent treatment of cancers, investigators face several challenges, including rapid cancer progression and invasion in healthy tissue, metastasis, and tumor recurrence [3].

Tumor cells have a number of molecular and physiological features that strongly distinguish them from healthy cells of the body [4,5]. Such features include the following:

1. The maintenance of proliferation signals: One of the most important features of tumor cells is the ability to continuously proliferate uncontrollably. Cancer cells are capable of amplifying growth factor receptor genes, which allows them to enhance proliferative signals in comparison with normal cells [6,7].
2. Reduction in the effect of growth inhibitors: Multiple mutations in tumor suppressor genes (p53 is amongst the most well known) lead to the disruption of their biological functions, which allow tumor cells to avoid the intracellular regulation of cell proliferation [8].
3. The ability to avoid programmed cell death: Tumor cells develop various mechanisms to evade both external and internal inducers of apoptosis [9].
4. Immortalization of the tumor clone: Acquired telomerase activity allows cancer cells to bypass the natural limitation in the number of cell divisions due to the length of telomeric sections of chromosomes.
5. The induction of angiogenesis: Tumor cells are able to synthesize a number of molecular factors that allow the activation of angiogenesis in the tumor. The resulting vascular network has a number of specific characteristics that allow not only an increase in the metabolic supply of the tumor, but also contribute to the extravasation of tumor cells with their further metastasis [10,11].
6. Invasion and metastasis: Metastasis is a complex multi-step process that allows tumor cells to spread throughout the body [12]. It depends on changes in the adhesive properties of tumor cells and also requires constant remodeling of the cytoskeleton.

The potency towards the spontaneous spreading of tumor cells into normal tissue is what makes gliomas such difficult cases for diagnosis and therapy. Upon oncotransformation, cells acquire movement mechanisms not observed in cells of the native tissue, which also serves as the source of tumor development, together with specific interaction with the extracellular matrix (ECM) [13]. This movement and ECM fixation is the basis for tumor invasion into surrounding organs and tissues, with the further development of the mitotic process, thereby causing tumor progression. The main features of changing the migration characteristics of tumor cells are an increase in the speed of cell movement, as well as a greater contribution of random wandering compared with healthy mobile cells of the body [14]. The types of tumor cell mobility are also very variable, which hints at the heterogeneity of regulating protein complexes [15].

In migration and metastasis, the molecular mechanisms will be similar: dynamic changes in the cytoskeleton, various molecular interactions between tumor cells, the microenvironment and the ECM, as well as the influence of various microRNAs. Interestingly enough, most of them can be linked to the chaperone family of heat shock proteins (HSPs). HSPs play a pivotal role in cancer progression by stabilizing oncogenic proteins, which allows cancer cells to resist stress and evade apoptosis, resulting in more aggressive tumors that are less responsive to treatments like chemotherapy. They also facilitate metastasis through proteins like HSP90 and HSP70, which help cancer cells survive migration challenges and promote epithelial–mesenchymal transition (EMT), enhancing their invasive capabilities. Despite their protective role, HSP-targeted therapies, including inhibitors of HSP90, HSP70, and HSP27, are under investigation in order to disrupt their cancer-supporting functions [16,17,18]. Researchers are also exploring HSP-based vaccines and the potential of exploiting overexpressed HSPs for targeted therapy delivery. Balancing the inhibition of HSPs while maintaining their essential functions in normal cells remains a critical challenge for future cancer therapies. However, the emerging understanding of the heat shock protein system as a network of interacting proteins dictates the necessity of wider (or even -omic) approaches [19]. This is where the detailed study of proteins supporting and regulating HSPs—co-chaperones—may play a pivotal role in developing more specific and sensitive diagnostic and therapeutic approaches. At this time, authors have already reported on cancer-specific HSP network formation. The aim of this review is to describe the current key members regulating HSP70 and HSP90 machinery, and illustrate via the most recent studies, how these proteins cooperate and contribute towards neoplasm progression.

## 2. Molecular Chaperones

The term molecular chaperone was first used in relation to the emerging understanding of protein topology and folding phenomena in 1968, when previous electrostatic-based theories were proven to be insufficient [20,21].

This heralded years of scientific research regarding cell proteostasis, now viewing families of constitutive and stress-induced chaperones working as a network [22,23,24]. Differences in structure and localization dictate a robust variety of chaperones, but the heat shock protein system has been the most widely researched in various physiological conditions [25,26,27,28,29,30,31]. Currently, several major HSP families are annotated based on structure and gene homology, including HSPH (HSP110), HSPC (HSP90), and HSPA (HSP70) [32,33,34].

Heat shock proteins play a multi-faceted role in the development of cancer, contributing to the survival and growth of malignant cells. In particular, they suppress apoptosis, regulate necrosis, bypass cellular aging, interfere with antitumor immunity, stimulate angiogenesis, and support metastasis [35,36].

HSPA1A (Hsp70), in particular, is actively involved in suppressing apoptosis, programmed cell death, which is one of the key mechanisms of the body’s defense against cancer cells [37,38,39]. Prolonged exposure to HSPA1A can lead to the development of immune tolerance, which promotes tumor growth [40,41]. At the same time, complexes of peptides and HSPA1A in low doses can stimulate antitumor immunity [42]. In addition, HSP70 plays an important role in stimulating angiogenesis, that is, the formation of new blood vessels that are necessary for nutrition and tumor growth [43,44].

The molecular structure of many HSPs is characterized by the presence of several functionally significant domains that contribute to its chaperone activity and participation in various cellular processes [45]. HSP70 consists of an N-terminal ATPase domain (NBD), a substrate-binding domain (SBD), and a C-terminal domain (CTD) interconnected by linker regions, all of which cooperate and contribute to client protein recruiting and folding [46,47].

Of particular interest is the role of the lipid environment in the functioning of HSPA1A [48]. It has been shown that HSPA1A is able to be translocated to the cell membrane (mHsp70 form). This membrane localization complicates detection by conventional methods, thus necessitating the development of novel approaches, which can detect both free and lipid-bound Hsp70, especially in non-small cell lung carcinoma (NSCLC) and glioblastoma patients [49]. Recent studies have continued the investigation of membrane HSP variants in the tumor context. In addition, Hsp70 can be released from cells in a membrane-associated form, for example, as part of exosomes [50].

### 2.1. Chaperone Cycles

**Figure 1:**
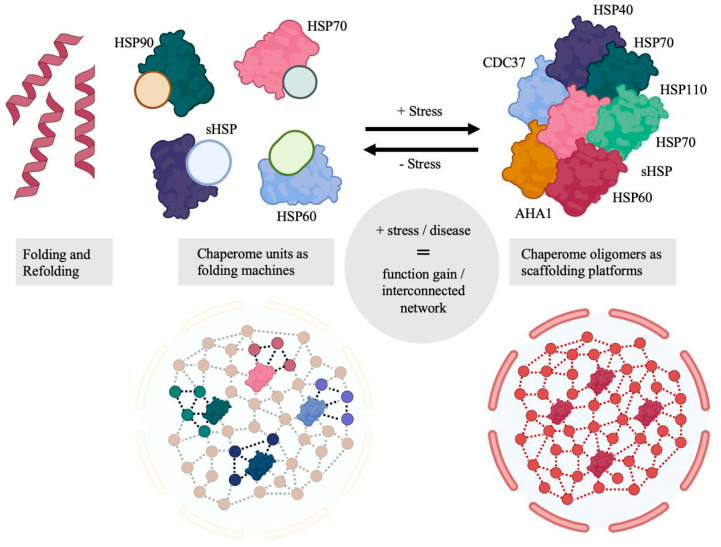
Functional gains from formation of multimeric chaperome scaffolding platforms under cellular stress. Adapted from [58]. Conceptual schematic; no quantitative data shown.

Though capable of a variety of protein–protein interactions, chaperones are largely unable to perform basic housekeeping functions individually. Instead, representatives of chaperone classes are combined with effector proteins: nucleotide and substrate exchange factors, adapter proteins, signal sequences, etc. The set of chaperones in this functional complex was termed “chaperome” in 2006, and, when a system considered is not limited to chaperones and has a high-order structure, an “epichaperome” [51]. In addition to the epichaperome’s presence in the cytoplasm of tumor cells, recent studies indicate that the chaperone cluster (including members of the HSP70 and HSP90 families) is also present on the surface of the plasma membrane, likely forming a functional epichaperome complex, with inhibitory analysis revealing its involvement in cancer cell invasion and migration in brain tumors (including primary glioblastomas from adult neuro-oncological patients) [52,53]. In 2013, the chaperome described the cooperative function of all previously mentioned HSP families, as well as their non-chaperone regulators [54,55]. By 2016, advanced bioinformatic research had expanded the list to 332 entries, represented by 88 chaperones (27%), of which 50 were ATP-dependent, and 244 co-chaperones (73%) [56,57]. Based on such an expanded network, it has become possible to build models of diseases associated with protein folding disorders [58]. Moreover, the chaperone was now presented as a wide network of interconnected proteins with several functional nodes that differ in their role in proteostasis and localization in cell compartments (Figure 1).

#### 2.1.1. HSP70 Regulation

One of the most represented and studied chaperome nodes are proteins of the HSP70 family, constitutive HSPA8 and stress-induced HSPA1A, accounting for up to 2.7% of the mass of cell proteins [59]. Both these proteins exhibit a great variety of functions and on-site forms according to cellular state [60,61]. In addition to localization, specialized homologues differ in their sites of substrate recognition and the degree of coordination with subnetworks of other chaperones, for example, HSP90 and HSP110 [62].

**Figure 2:**
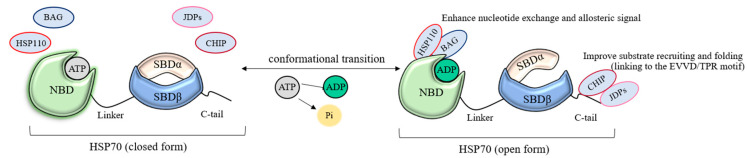
Schematic presentation of the HSP70 chaperone cycle with key regulator proteins. Conceptual schematic; no quantitative data shown.

The chaperone cycle of HSP70 is based on the dynamism of their two-domain structure: the binding and hydrolysis of ATP on the nucleotide-binding domain (NBD) provokes a change in the topology of the protein molecule and transmits an allosteric signal to the substrate-binding domain (SBD), changing the affinity towards polypeptide chains (Figure 2) [63]. A significant part of the regulation of the HSP70 cycle takes place precisely at the nucleotide-binding domain with the help of nucleotide exchange factors (NEFs). Several notable proteins affect HSP70 function, while also participating in various cellular mechanisms [64].

Bag, HSP110 (HSPH), and Armadillo proteins function in the cytosol and on the plasma membranes to stabilize the “open” conformation of the chaperone. The interaction of the nucleotide-binding domain of HSP110 with the similar domain of other HSP70 proteins (based on yeast homologues, Sse1 and Ssa1) gives them a mutual position that catalyzes the exchange of nucleotides and their hydrolysis [65,66]. Armadillo and Bag have conservative binding sites, affecting the conformation of only certain subdomains of HSP70. The effect of the catalysis of the ATP cycle of HSP70 in these families differs within an order of magnitude. Perhaps this is what characterizes the different dynamics of the chaperone cycle in the processes of proteostasis, and it is the NEF that controls the course of folding and disaggregation. It is worth noting that the role of these proteins is not limited to catalysis: some of them have additional recognition sites that allow HSP70 to recruit certain substrates or direct their localization after folding, and even combine chaperone complexes into networks [67].

The regulatory and substrate-recruiting function in the HSP70 node is performed by J-domain proteins (JDPs) of different types (by structure) [68]. The J-domain is able to bind to the NBD and SBD HSP70 contact sites due to ionic and hydrophobic interactions, significantly enhancing allosteric signal during ATP hydrolysis [69]. JDPs binding to the EEVD motif of HSP70 open the way to the binding of misfolded polypeptide chains for their subsequent refolding, though this effect is not exclusive of non-EEVD-dependent JDPs [70,71]. By aligning the conformation of the substrate with the surface of their domains, these co-chaperones “choose” the optimal one for HSP70 from the set of possible conformations in equilibrium [72]. Due to the small recognition site, JDPs do not exhibit specificity, and their affinity is governed by JDP and surrounding linkers [73,74]. The analysis of mutant JDP variants also showed that these proteins have two conservative substrate recognition sites on the β-surfaces of the C-terminal domain, enhancing protein binding and folding on SBD [75,76]. Studies of mutant JDP deprived of the Zn^2+^ binding site have shown that while maintaining affinity for the substrate, the ability of JDP to subsequently dissociate it and transfer constitutive HSPA1A is lost [77]. Considering the above-described features of J-domain proteins, it can be concluded that this class of proteins is one of the key activators and regulators of the chaperone node for HSP70, playing an equivalently important role both in folding synthesized polypeptide chains and in maintaining protein aggregates for their refolding. The study of the characteristics of individual JDPs, as well as their intra-class cooperation and interaction with other components of the chaperome, will reveal the key mechanisms responsible for cell proliferation.

The CHIP protein, common to several chaperone families, also competes for binding with HSP70 via EEVD and TPR motifs [78]. It is able to label proteins with a damaged conformation binding to HSP70 for their subsequent degradation in proteasomes. It is worth noting that the CHIP-HSP70 complex needs a Bag protein again, this time to transfer the substrate to the proteasome [79]. It has also been experimentally shown that CHIP, like chaperones, is prone to overexpression under cellular stress [80]. Thus, this protein is the main regulator of protein degradation in chaperome nodes.

Recent insights deepen our understanding of HSP70’s environment of interactors. First of all, the chaperone can be integrated into various dynamic functional complexes depending on the set of cellular events. In these complexes, several proteins play the role of adaptors for HSP interactions, while others dictate specific functions via NEFs and substrate affinity control. Conservative domains on both participants are integral for complex stability and equilibrium. It may be suspected that rewiring these HSP nodes towards GTPase activation and cytoskeleton support plays a major role in facilitating tumor cell motility.

#### 2.1.2. HSP90 Regulation

Chaperones of the HSP90 family are also among the most widely represented in the cell and are responsible for the maturation and maintenance of protein stability, their degradation, and the maintenance of cell signaling pathways [81]. Some members of the family have specific localization, but the majority of HSP90 functions in a dimer state in cytosol [82,83]. Overexpression on malignant neoplasm in a similar way to HSPA1A has made HSP90 and related protein networks an important object of research in molecular oncology.

**Figure 3:**
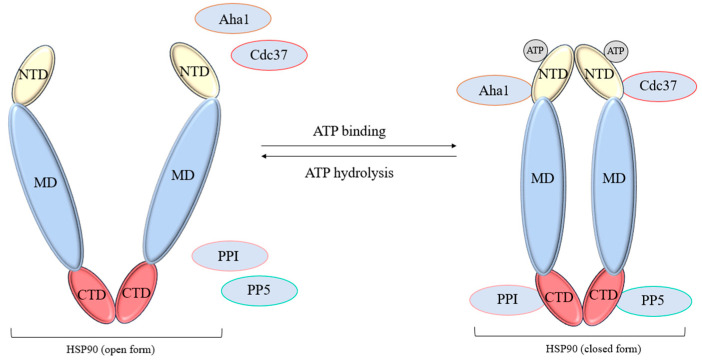
Schematic representation of HSP90 chaperone cycle with key regulator proteins. Conceptual schematic; no quantitative data shown.

The HSP90 chaperone has characteristic homologous C- and N-ends (CTDs, NTDs) and a middle domain (MD), which are connected by disorganized linkers. Linkers allow the chaperone to change conformation and transmit an allosteric signal similar to HSP70; however, a negatively charged linker is found in eukaryotes, additionally binding NTDs and MD [84]. Other notable differences include specified dimerization sites and a “spare” nucleotide-binding motif on CTD [85]. The middle domain of the protein mainly serves to bind substrates and transmit an allosteric signal during the chaperone cycle (Figure 3).

HSP90 participates in proteostasis immediately after the completion of the HSP70 cycle, completing protein folding and directing it to transport to the required compartment, which requires the assembly of a functional HSP70-HSP90 complex. One of the key regulators of this complex is the multidomain protein HOP, binding to the EEVD/MEEVD sequence through TPR [86]. Due to the ability to simultaneously and ATP-independently bind the HSP70 and HSP90 dimers, HOP is considered an adapter co-chaperone [87,88].

Upon the successful transfer of the substrate from one chaperone to another, the HSP70-HSP90 complex is disassembled, releasing HOP, which also entails conformational changes in the participating proteins. However, it is also worth noting that, like most chaperone networks, the HSP90 node has redundancy and dynamism. Studies on yeast cells have shown that the knockdown of such an adapter protein did not serve as a critical change in proteostasis—presumably, the chaperone can adapt and the transfer of the substrate from HSP70 to HSP90 can occur directly [89].

Another important class of co-chaperones for HSP90 are peptidyl-prolyl isomerases (PPIs). They are represented by two structurally unrelated groups of proteins—these are cyclophilins (Cyps) and the FKBP family (tacrolimus-binding proteins). Both groups also bind exclusively to the MEEVD site of HSP90 [90]. The PPI domain in the cotranslational regulation of the newly synthesized polypeptide chain makes the substrate more accessible to chaperones [91]. Moreover, some representatives of this group of proteins are necessary for the membrane translocation of synthesized polypeptides [92]. For FKBP52, it was found that binding to HSP90 allows the FK domain to interact with the ligand-binding domain of the glucocorticoid receptor (GR), which serves to anchor substrates in the chaperone complex [93].

No similar GR stimulation was found for mutant versions of FKBP52 and for native FKBP51 [94]. A separate fact establishing the role of some immunophilins in protein traffic was the presence of a connection with the dynein complex [95]. Thus, an attempt was made to explain the activation of substrate-binding receptors without disrupting the dimerization of HSP90. The system is called the “transportosome” and assumes the possibility of retrotransportation of many soluble proteins. However, due to structural differences and the excessive presence of immunophilins in the chaperone, many representatives remain poorly studied.

Another TPR-dependent co-chaperone HSP90 is Pt1/PP5 phosphatase. In the native form, the enzyme domain is blocked by tetratricopeptide sites using a network of hydrophobic interactions [96]. Upon contact with the MEEVD site of HSP90, the auto-inhibited protein is activated, acquiring serine–threonine phosphatase function. In connection with HSP90, PP5 is directly involved in the regulation of cell signaling pathways. One of the most studied pathways involving HSP90 is the RAF-MAPK cascade, which regulates cell proliferation. It relies on the activation of growth hormones through phosphorylated forms of RAF and RAS GTPases. In this case, PP5 activity is necessary to inhibit signal transmission [97].

Of the TPR-independent HSP90 co-chaperones, several are of particular interest in the chaperome. The Aha1 protein is the only one of them capable of catalyzing the hydrolysis of ATP to NBD. Here, the binding is mainly due to the MD sites of HSP90, without relying on the MEEVD motif [98]. This leads to conformational changes in the NBD subdomains responsible for nucleotide binding and hydrolysis. It is also assumed that in the dimeric form of HSP90, both ATP and Aha1 provoke conformational changes in a different order in both chaperones, accelerating the transition of HSP90 forms during the functional cycle [99].

From a structural point of view, the key co-chaperone Sgt1 for the assembly of protein complexes behaves in a similar way. Despite the presence of the TPR domain, this protein selectively binds to the NBD of HSP90 [100]. At the functional and genetic level, it has been proven that in the chaperone cycle Sgt1 is an adapter for connecting chaperones with enzyme complexes and some substrates necessary in proteostasis [101].

On the other hand, a specific adapter is required to maintain signaling pathways independently of the MEEVD motif of the HSP90. This is the co-chaperone Cdc37, which has a high affinity for the cell kinome. Similarly to the GR method, Cdc37 is able to sequentially bind to partially unfolded kinase sites, “sorting” the substrates available to the chaperone [102]. Cdc37 substrate transfer requires recognition of the closed, Aha1-stimulated conformation of the HSP90 dimer [103]. As shown, the inhibition of any participant in this chaperone complex leads to the disruption of the synthesis and maturation of key kinase signaling pathways [104].

The interactions of the functional HSP90 dimer are mostly embedded in signaling pathways utilizing GTPases, a necessary step for pseudopodia formation and thus, tumor cell migration. Since the participants in the HSP90 cycle rely on both the TRP domain and specific recognition sites, they can provide varying responses to stimuli in cancer, aligning biological processes towards tumor progression.

## 3. Tumor Cell Motility and Invasion

Tumor cells have a distinct type of movement which is not usually found in healthy cells before oncotransformation. The main features of change in the migration characteristics of tumor cells would be an increase in the speed of cell movement, as well as a greater contribution of random wandering compared with healthy mobile cells of the body [105,106]. The types of movement characteristic of healthy motile cells of the body can also be observed in a tumor; they include amoeboid, mesenchymal, epithelial, collective, and individual movement. This variability indicates a set of molecular mechanisms that coordinate the movement and invasion of tumor cells. Two key consequential processes involving tumor cell motility are known to initiate metastasis:

1. Invasion. Cancer cells are capable of performing a pathological epithelial–mesenchymal transition (EMT). This process occurs due to an increase in the level of contractile activity of actomyosin and non-apoptotic blebbing activity, which allows the cell to move in the already-existing pores of collagen networks with minimal adhesion and matrix remodeling [107,108].
2. Intravasation. A process in which single tumor cells or groups of them disrupt the integrity of the basement membrane of a vessel, thereby penetrating into its lumen. It is important to note that the vascular network, the development of which was induced by the tumor, has a number of features contributing to the process of intravasation, namely, the presence of accessible points for the penetration of tumor cells into the vascular wall, sufficient vessel lumen for the passage of both individual cells and their groups, and sufficient blood flow velocity, contributing to the removal of intravasated cells [109,110].

The molecular mechanisms of tumor cells include dynamic changes in the cytoskeleton, various molecular interactions between tumor cells, the microenvironment, and the ECM, as well as the influence of various microRNAs. All these mechanisms contribute to the progression of cancer, either via single cell migration or stream-like and organized group invasion through healthy tissue. The mode of cancer cell invasion is found to be largely dependent on two major proteins: E-cadherin for cell–cell connection, and integrin beta-1 for cell–matrix adhesion [111]. Collective invasion in the later stages of tumor development required the dynamic reorganization of adhesive contacts and surrounding matrix, thus, the expression levels of cadherin and integrin significantly change with cancer progression, and the decreased production of these proteins has been found to be a marker for active metastasis.

As for the movement of the cancer cell and cellular structures, proteins of small GTPase type have been widely researched due to their involvement in membrane and cytoskeleton dynamics. Key GTPase subfamilies, Rho, Rac, and CDC42, exhibit increased expression in various types of cancer, especially in late-stage solid tumors [112,113,114].

The activity of abundant Rho GTPases, such as RhoA, Rac1, and Cdc42, is regulated through a cyclic transition between inactive GDF-bound and active GTP-bound states. This process is controlled by guanine nucleotide exchange factors (GEFs), which promote activation by stimulating the exchange of GDP for GTP, and proteins that activate GTPase activity (GAP), which initiate inactivation through the catalysis of GTP hydrolysis. Dynamic regulation requires the coordinated and localized operation of multiple components. In contrast to typical ones, some atypical members of the Rho family, such as the Rnd and RhoH subfamilies, are predominantly in the GTP-bound form [115].

Small GTPases of the Rho family are key regulators of the actin cytoskeleton and, as a result, play a central role in cell motility and invasion [116,117]. They mediate the formation of cellular outgrowths such as filopodia and lamellopodia by regulating actin polymerization through various effectors, including formins (mDia1/2) for RhoA and the Arp2/3 complex through N-WASP and WAVE for Cdc42 and Rac1, respectively. RhoA activity is mainly observed in the posterior part of migrating cells, contributing to the contraction and tightening of the cell body, while Rac1 is most active at the forefront, participating in the formation of protrusions. The spatiotemporal regulation of RhoA and Rac1 activity is strictly controlled, including mechanisms of mutual inhibition [118].

RhoA plays a significant role in the motility and invasion of glioblastoma cells. The inhibition of Rho kinase, one of the RhoA effectors, affects astrocytoma morphology, motility, and invasion by activating Rac1. Resveratrol suppresses the migration and invasion of human glioblastoma cells through the activation of the RhoA/ROCK signaling pathway [119].

RhoA participates in the formation of mature focal-type adhesive contacts and actin stress fibrils, providing the generation of contractile forces in the posterior part of the cell necessary for movement. RhoA also interacts with diaphanous formins (mDias), initiating actin polymerization. The inhibition of Rho kinase affects astrocytoma morphology, motility, and invasion through the activation of Rac1 [120]. Rac1 initiates actin polymerization at the leading edge of the cell, leading to the formation of lamellopodia and membrane folds, which is necessary for the displacement of the cell membrane in the direction of migration [121]. The expression level of Cdc42 also correlates with the malignancy of human glioma. Cdc42 is mainly involved in establishing cellular polarity and initiating the formation of filopodia, thin cytoplasmic outgrowths that serve to sensitize the microenvironment and determine the motion vector [122]. Cdc42 transmits signals from the extracellular medium to effector proteins, determining the orientation of the cell. Cdc42 is able to activate Rac1 [123].

Of course, tumor cell motility is not dependent on Rho activity alone, and recently many Rho-interacting proteins and other GTPases have been researched in terms of cancer progression. What is interesting is that both Rho and HSP family proteins, being highly expressed in solid tumors, are interwoven in proteostasis, and may be incorporated into a larger interactome network. Investigating the links between GTP-associated migration and HSP activity may bring to light tumor progression hubs for multi-faceted diagnosis and therapy.

## 4. GTPase and Motility Effectors Within Chaperone Network

It has long been discussed that an interconnected network of HSP effector and client proteins may form upon tumor cell transformation—the epichaperome—and since it includes proteins involved in various signal pathways, this network facilitates a higher rate of tumor cell motility and invasion.

Analyzing the interactions within chaperome proteins, we can reveal a palette of cell migration hubs. For instance, the studies in [124] report that a peculiar protein complex is formed during the HSP70 functional cycle, where FAF1, a scaffold protein, both supports ubiquitin-related processes in tumor formation and participates in cell-to-cell adhesion. It was found that the N-terminal region of FAF1, in particular the UBL1 domain, binds to Hsp70. This FAF1-Hsp70 complex serves as a framework that facilitates the interaction of FAF1 with key cytoskeletal organizers, primarily IQGAP1. IQGAP1 is known to play the role of a negative regulator of RhoA-GTPase, and we assume that this interaction is central to the ability of FAF1 to suppress RhoA activity. Consequently, the loss of FAF1 leads to the hyperactivation of the RhoA-ROCK-MLC2 signaling pathway, which, in turn, leads to the excessive accumulation of actomyosin stress fibers. This restructuring of the cytoskeleton underlies the observed morphological changes: an increase in cell size and a shift towards a more spherical shape. In addition, the abnormal activity of RhoA disrupts the normal distribution of β-catenin in the cytosol, which ultimately leads to a violation of the integrity of adhesive compounds. Moreover, the use of the ROCK inhibitor Y-27632 in cells where FAF1 expression was suppressed (and the FAF1-HSP70 complex) inhibited the disassembly of the adhesive compound by suppressing pMLC induction, which means that the ROCK inhibitor eliminates the disruption of the adhesive compound caused by FAF1 knockdown. These results suggest that FAF1 depletion degrades the adhesive bond due to RhoA activation, and then increases the formation of stressed fibers due to ROCK activation.

In another study, a new model was proposed representing the interaction of HSP70-1 and CK2β [125]. It was found that HSPA1A interacts with the non-catalytic subunit of the CK2 -CK2β protein kinase, but HSPA1A itself does not act as a CK2 substrate or activity modulator, which suggests that it is probably an interaction partner with free CK2β or CK2β, as part of the CK2 holoenzyme. The authors suggest that the interaction between CK2β and HSPA1A in human prostate carcinoma cells has a stabilizing effect on free CK2β, which is rapidly ubiquitinated and decomposed in the cell. This interaction may be due to the fact that HSPA1A stabilizes CK2β as a chaperone by regulating the presence of unbound CK2β in the CK2 holoenzyme. The enhanced interaction after heat shock confirms the assumption that HSPA1A prevents the heat-related decomposition of CK2β followed by aggregation or degradation. At the same time, a large number of proteins involved in the reorganization of the cytoskeleton as partners were identified in the CK2b interactome. In particular, the leukemia-associated Rho-GEF (LARG), which is involved in migration and the cell cycle, is of great interest. This study suggests a CK2β-mediated interaction between HSPA1A and the Rho signaling pathway.

**Figure 4:**
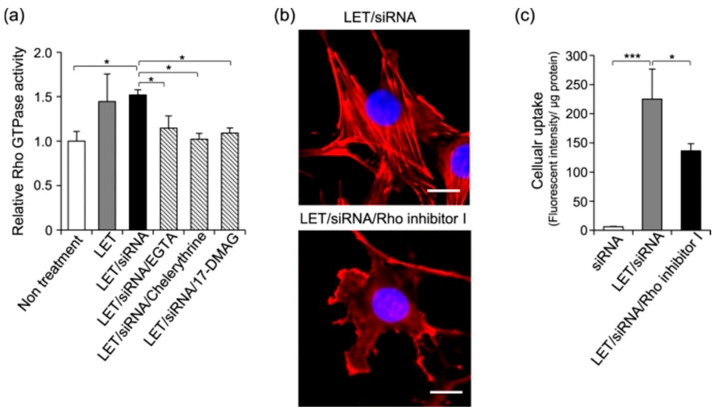
Activation of Rho GTPase by LET with siRNA. (**a**) Relative Rho GTPase activity. After 36 h of serum starvation, cells were treated with LET in presence of siRNA, and were lysed immediately after LET. Rho GTPase activity in the lysate was measured by G-Lisa activation assay (Cytoskeleton Inc., Denver, CO, USA). (**b**,**c**) Cells pretreated with Rho inhibitor I for 4 h, followed by LET in presence of siRNA. (**b**) Effect of Rho inhibitor I on actin cytoskeleton remodeling induced by LET with siRNA. Red and blue signals indicate rhodamine phalloidin-labeled actin and nuclei, respectively. Scale bars indicate 20 μm. (**c**) The effect of Rho inhibitor I on LET-mediated cellular uptake of siRNA. Values represent the means of 3 individual experiments. Bars represent standard deviations. * *p*  <  0.05 and *** *p*  <  0.001 [126].

In a similar fashion, protein kinase C (PKC) is linked to the activity of the HSP90 chaperone. Mahadi et al. performed a physiological study of low electric treatment (LET) on 3T3 cells, revealing the interplay between siRNA uptake and Ca ion influx [126]. They observed upregulated levels of HSP90 and its co-chaperones and interactors (including the PKC substrate) upon cell treatment, as well as stimulated chaperone activity. This activity specifically enhanced the cellular uptake of various macromolecules, confirmed by an inhibitor study. Concurrently with macromolecule uptake, the interplay between PKC and HSP90 activation was also found to stimulate Rho GTPase and therefore induced actin cytoskeleton remodeling that involved the formation of lamellipodia and associated stress fibers (Figure 4). This phenomenon may display a peculiar link between cancer cell communication and motility stimulation through the molecular environment of chaperones.

Another HSP70 interactor is directly involved in PKC function—RACK1—which is found to be overexpressed in various types of tumors. The authors of [127] describe the contribution of RACK1 to tumor growth in the initial stages of prostate cancer, confirmed by siRNA assays. The effect is due to AKT mediation and p53 suppression, and AKT cascade is known to facilitate tumor cell motility. Additionally, for breast cancer it was recently shown that abundant RACK1 mediates the tumor growth in vitro and in vivo due to its involvement in the WNT pathway and β-catenin [128]. Described as a partner for RACK1 and intersecting with the HSP70 interactome protein K2C1 completes the linking of the PKC-RACK1 complex with tumor cell migration through the ITGB1 molecular environment [129].

The authors’ results in [130] additionally characterize the connection of the HSP70 chaperome with proteins of the Rho family, this time through the ARHGEF10L gene. The protein encoded by the ARHGEF10L gene belongs to the family of guanine nucleotide exchange factors (GEFs), including for Rho signaling. This protein is known to be overexpressed in some types of cancer, such as colorectal cancer, cervical cancer, and glioma. It was found that HeLa cells transfected with plasmids expressing ARHGEF10L showed significantly increased cell proliferation within 48 and 72 h according to the CCK-8 test. Also, the results of the wound healing test showed the increased migration activity of such cells. Moreover, the overexpression of ARHGEF10L has been shown to activate the GTP-RhoA-ROCK1-pERM pathway in HeLa cells. In addition, RNA sequencing of SGC7901 gastric cancer cells overexpressing ARHGEF10L revealed an increased expression of HSPA6, while no such effect was observed in cells that were transfected with empty plasmids. The authors suggest that the overexpression of ARHGEF10L most likely contributed to the proliferation, migration, and invasiveness of tumor cells by stimulating HSPA6 expression, or they are coexpressed.

J-domain proteins involved in the HSP70 cycle have many known connections to cell migration. For instance, A-type (DNAJA1,3) and B-type (DNAJB4,6) protein expression has been shown to negatively affect tumor cell migration [131,132]. However, during the chaperone cycle these proteins are allocated to curating HSP70 substrate binding, which may lower the pool of available JDP and thus mitigate their inhibitory effect. In fact, recent studies show that DNAJA1 (Hdj2) binds with mutant p53, affecting cancer cell proliferation and metastasis. Kaida et al. [133] performed an investigation of mutant and native p53 and DNAJA1 dynamics. When comparing the JDP levels in HNSCC to normal tissue cells, authors reported high DNAJA1 accumulation in tumors irrespective of the carried p53 type. Interestingly, the knockdown of either DNAJA1 or mutant p53 had similar effects in lowering filipodia formation, with the full knockout of these proteins further enhancing the effect. Further studies revealed that this is due to the lowered functional activity of CDC42 and RAC1 GTPases. This interaction was present only for the conformational p53 mutant, as the knockdown of DNAJA1 on wt. or null p53 cells had minimal effect.

Interestingly, DNAJA depletion exhibited drastically different results in glioblastoma cells. Employing lentiviral-mediated short hairpin RNA (shRNA) interference, stable knockdown cell lines targeting HSPA1A, DNAJB1 (Hdj1), or DNAJA1 (Hdj2) were established [134]. Protein depletion was verified by Western blotting, with reductions of approximately 87% for HSPA1A, 92% for DNAJB1, and 53% for DNAJA1. C6-shHdj2 cells became rounder with diminished adherence, increased floating cell fractions, and had multiple leading edges indicative of altered motility. These cells had higher invasiveness in a transwell model and exhibited ameboid-type movement. Conversely, C6-shHsp70 cells adopted an elongated, fibroblast-like appearance and demonstrated slower proliferation rates, in agreement with the canonical pro-survival and growth-promoting functions of HSPA1A. Additionally, DNAJB1 depletion led to a mislocation of cadherin, impairing cell adhesion, and increased levels of MMPs according to zymographic analysis. MRI monitoring of in vivo models yielded unexpected results: DNAJA1 knockdown markedly accelerated tumor growth and augmented malignancy, as evidenced by rapid progression, the emergence of leptomeningeal metastases, and a significant reduction in the lifespan of tumor-bearing animals relative to controls.

DNAJ3 on the other hand, has been recently found to be structurally more similar to B-class JDPs. An in-depth structural analysis of Tid1 [135] has shown its consistent conformational analogy to DNAJB1, and a stable interaction with GF-motif. Here, DNAJ3 mainly plays a role in mitochondrial maintenance. However, this JDP knockdown is associated not with decreased tumor cell proliferation or mitochondrial damage, but an increased rate of cell migration [136]. After deep investigation, the authors suggest that this is due to the increased activity of galectin-MMP9 which is directly integrated into the HSP70 interactome, since no ROS involvement was examined. Not only did the galectin-MMP9 pathway axis exhibit increased expression upon DNAJ3 knockdown, it also contributed to poor diagnosis in patients with examined gastric cancer types. The increased activity of MMP here can contribute to matrix remodeling, and, as such, epithelial–mesenchymal transfer and cell invasion.

In the HSP interactome, many co-chaperones act in assembly when recruiting different client or regulatory proteins. An important apoptosis regulator, BAG2, is known to co-dependently activate with the DNAJ-PKA complex in protein folding and signal transduction. Using proximity proteomics, researchers confirmed a tight interconnection between DNAJ-PKA, BAG2, and Stip1 in carcinoma cells [137]. The network proteins contributed to RNA processing as well as collagen biosynthesis and cytoskeletal remodeling—processes responsible for cancer cell migration and metastasis. Further experiments with dislocated PKA interactors using mutated DNAJ and PKA have shown that DNAJ-PKAc is not confined within AKAP signaling anchors and exhibits higher mobility, due to the disrupted association of the fusion protein with PKA regulatory subunits. The association with HSP70, in this case, is a contributing element for PKA diffusion in cytosol, which was linked to the increased chemoresistance of carcinoma cells.

Within the chaperone network, ATPase domain activators DNAJB/DNAJC bind to small GTPase protein DNAJC27 (RBj). Recent studies have shown an involvement of RBj in the MEK/ERK signaling pathway, where elevated levels of activity in this signal axis are involved in epithelial–mesenchymal transition. The authors of [138] analyzed lung cancer cells and confirmed increased levels of RBj expression. The knockdown of this protein impaired the growth, invasion, and migration of NSCLC cell lines by inhibiting the MEK pathway and thus, EMT. The overexpression of this DNAJC yielded the opposite results in vitro and was concurrent with accelerated tumor formation in vivo in an A549 nude mice model.

The study by Pare et al. investigates the role of the protein sacsin in the cell adhesion of neurons [139]. Sacsin, a large protein with a ubiquitin-like domain, interacts with several heat shock protein chaperones and is crucial for transporting alpha integrins to the plasma membrane. It is involved in protein chaperoning and quality control, similar to HSP90 and HSP40. The SacsJ-domain binds to proteins of the HSP70 chaperone family for the resorption of aggregates formed from GFP-ataxin-1 in SH-SY5Y cells. Sacsin affects the distribution of Rab1b GTPase, causing Rab1b to be concentrated in soma rather than over soma and dendrites. Additionally, the expression of neuroplastin, a key adhesion molecule in neuronal membranes, is reduced in Sacs-/- cells, suggesting a possible role of heat shock proteins HSP90 and HSP40 in the distribution of small GTPases and cell adhesion processes.

For HSP90 dynamics, a recent inhibitor study has shown several effects regarding the Aha1 co-chaperone. The authors report that the studied inhibitor—Benzbromarone—is a potential instrument for antitumor drug development, as it binds to HSP90-NTD and Aha1-CTD. This specific binding disrupts the interaction between chaperone and co-chaperone, impairing parts of proteostasis, which destabilizes HSP90 client proteins and induces cell apoptosis when tested in vitro on sarcoma and colorectal cancer. In both of these cell lines, the disrupted interaction also inhibited the rate of cell motility and invasion in vitro, which can be linked to dysregulated EMT proteins [140].

The authors in [141] were able to determine the role of MAPK signaling in the progression and therapy of cSCC by applying photodynamic therapy modified with 5-aminolevulinic acid (M-PDT). It was shown that this approach inhibits cell proliferation through the phosphorylated forms of three key MAP kinases, namely through one of the cascade participants: the suppression of p-Erk1/2 and the enhancement of p-JNK and p-p38. MAP-phosphatase 1 (MKP1) and serine/threonine protein phosphatase 2A (PP2A) are the main phosphatases that negatively regulate the phosphorylation of Erk1/2, JNK, and p38, and protein phosphatase 5 (PP5), an HSP90 co-chaperone, has been identified as a negative regulator of the JNK cascade, involved in the stress response. Thus, the authors showed that M-PDT inhibits cSCC cell proliferation by activating PP2A and inhibiting PP5, which leads to the inhibition of Erk1/2 and the activation of JNK and p38 pathways in cSCC, and as a result, the suppression of proliferation.

GTP cyclohydrolase (GCH1), which is not directly a co-chaperone but a prominent HSP-GTPase network member, is crucial for tetrahydrobiopterin (BH4) biosynthesis. The study in [142] reveals that GCH1 enhances the growth of triple-negative and HER2+ breast cancers and transforms nontumor breast epithelial cells. Notably, GCH1 is also associated with EMT due to binding to vimentin, independent of BH4 production, and is mediated by HSP90. Proteomic analysis confirmed network connectivity between this protein and chaperone cycles. GCH1 ablation reduces tumor growth and disrupts key signaling pathways while activating p53. Higher GCH1 levels are associated with lower breast cancer survival, indicating an enzyme-independent oncogenic role and positioning GCH1 as a potential therapeutic target.

Extracellular HSP90 secreted by cancer cells may also affect tumor growth by activating substrate proteins and triggering signals through cell receptors. A previous study [143] identifies Morgana, an HSP90 co-chaperone, also released by cancer cells of various types, as a protein that facilitates cell migration. Silencing Morgana significantly impairs wound closure in motility assays. Differential antibody analysis revealed that proteins TLR2, TLR4, and LRP1 are involved in Morgana-mediated cell migration. In mouse cancer models, targeting Morgana reduced tumor growth through the macrophage-mediated recruitment of CD8+ T lymphocytes, inhibited migration, and minimized metastasis. These findings position Morgana as a key element in the HSP90 extracellular interactome, regulating HSP90’s role in cancer cell migration and antitumor immunity suppression.

RAF1 is a serine/threonine protein kinase, a regulatory component that supports the relationship between the membrane-bound GTPases of the Ras family and the MAPK/ERK signaling cascade. This key regulatory element acts as a molecular switch that determines cell fate, including the processes of proliferation, differentiation, apoptosis, survival, and oncogenic transformation. The expression of the Raf-1 proto-oncogene controls cell migration, apoptosis, and differentiation processes [144]. Recently, Nilanjan et al. described a unique position that RAF1 has within HSP70/HSP90 interactomes due to it being regulated by HOP [145]. In this study, in vitro assays suggested the physical interaction of HOP with CRAF and this co-chaperone’s involvement in kinase maturation. Utilizing the time-dependent overexpression of HOP in carcinoma cells, the authors confirmed RAF activation by accumulated intracellular HOP with MEK being a mediator protein. During this series of experiments, no HOP secretion or contribution of extracellular HOP was observed. Further experiments demonstrated the crucial role of HOP-HSP90 interaction through TPR domains in RAF mediation, as well as the peculiar facilitation of HSP90-actin interaction by a co-chaperone. Therefore, a novel MAPK mediating role is established for HOP in cell migration. Additionally, HOP has recently emerged as a critical molecule influencing several metastasis steps. Apart from its intracellular role, secreted STIP1 interacts with the cellular prion protein (PrPc) on tumor cell membranes. This interaction activates ERK1/2 signaling, which recruits integrins and cadherins to membrane sites, destabilizing existing adhesions and facilitating a more motile, less adherent phenotype. By enhancing the phosphorylation and stability of components within this signaling axis, STIP1 contributes to EMT and subsequent tumor cell migration and invasion. Silencing STIP1 expression reverses these effects, restoring epithelial characteristics and reducing invasiveness. In breast cancer, STIP1 localizes extracellularly with Hsp90 to stabilize matrix metalloproteinase-2 (MMP-2) [146]. The study by [147] focused on human prostate cancer (PCa) to investigate how cell stress affects HSP90α and MMP-2. Stress was induced in PCa cell lines through heat shock and serum starvation, with intracellular and extracellular proteins analyzed via Western blot. Surprisingly, increased levels of HSP90α under stress correlated with decreased MMP-2 activity, indicating a complex regulatory mechanism. HSP90α knockout PCa cells showed normal growth but reduced invasion, suggesting HSP90α’s role in modulating stress responses and MMP-2 activity. This research offers a novel understanding of the interplay between cell stress, secreted factors, and PCa cell motility, emphasizing the significance of HSP90α in these processes.

As an HSP90 regulator, CDC37 itself has an intricate link to RAF1 kinase. Recently, a stable full-length complex of RAF1-HSP90-CDC37 (RHC) was described [148]. In the description, an HSP90 dimer acts as a core, separating the N- and C- terminal domains of RAF while maintaining its conformational mobility, and supporting that CDC37 also interfaces with RAF domains. In vitro assays confirmed that disrupting this RHC machinery decreases cell proliferation. The interface between RAF1, CDC37, and HOP may represent a vulnerable spot for therapeutic strategies. Conversely, CDC37L1, sharing about 31% homology with CDC37, has been less characterized in the oncological context. The immunohistochemical staining of gastric cancer tissue microarrays revealed a marked decrease in CDC37L1 protein expression correlating with higher tumor grades in gastric cancer [149]. Overexpression experiments demonstrated that elevating CDC37L1 levels in GC cell lines significantly inhibited cell proliferation in various assays. The analysis of cell migration using transwell chambers indicated that CDC37L1 also suppresses GC cell motility. In vivo validation through xenograft models in nude mice affirmed these findings; GC cells overexpressing CDC37L1 formed smaller tumors with diminished growth rates compared with control cells. Silencing CDC37L1 notably increased CDK6 protein expression without significant changes in other related cell cycle proteins, indicating selective modulation. Therefore, unlike CDC37, which generally promotes oncogenic kinase activities and tumor development, CDC37L1 exhibits an opposing function by suppressing GC cell growth and motility through downregulating CDK6.

P23, a co-chaperone of HSP90, has been identified as a succinate-activated transcription factor that critically contributes to the tumorigenesis of lung adenocarcinoma [150]. Investigations reveal that within tumor cells, the metabolite succinate promotes the succinylation of p23 at specific lysine residues (K7, K33, and K79). This post-translational modification is essential for the nuclear translocation of p23, enabling it to bind to and activate the promoter of cyclooxygenase-2 (COX-2). The authors observe that the depletion of p23 via an RNA interference reduces lung adenocarcinoma cell proliferation, invasion, and colony formation, while the overexpression of p23 enhances these malignant behaviors in vitro and accelerates tumor growth in vivo. Succinate buildup in the tumor microenvironment triggers p23 succinylation and nuclear translocation, distinct from canonical inflammation pathways that activate COX-2 via cytokines like TNF-α, IL-1β, or LPS through NF-κB signaling. In the study, a small molecule M16 was identified. This compound potently inhibits p23 succinylation and DNA binding to the COX-2 promoter, effectively suppressing COX-2 transcription. In vitro, M16 significantly reduces tumor cell viability, proliferation, and migration, and contributes to apoptosis without affecting p23 protein expression, underscoring its specificity. These results add metabolic context to the previously established role of HSP90 in cancer cell proliferation.

## 5. Chaperome Members in Tumor Vesicle Trafficking

Apart from direct cytoskeleton remodeling, the contribution of HSP regulators towards filament dynamism may affect vesicular trafficking, in particular, endosome–exosome transfer. Exosomes reportedly play a role in cancer progression by facilitating communication between tumor cells and their microenvironment. Notably, they help in remodeling the extracellular matrix, preparing distant niches for metastasis, and modulating immune responses to favor tumor survival via transporting various enzymes and signal peptides [151,152]. Exosome biogenesis and secretion are regulated by complexes such as ESCRT, Rab GTPases, SNARE, and the syntenin-ALIX-syndecan axis. Heat shock proteins such as Hsc70 and HSP90 are shown to be integral in exosome formation, cargo sorting, membrane fusion, and stress response, interacting with Rab GTPases and ALIX to maintain vesicle trafficking and selective loading [153].

HSP90 assists Rab GTPase recycling via the GDI complex, ensuring sustained exosome function [154]. Hsc70 involvement in cargo sorting is linked to AP2 adaptor complex degradation, enhancing targeted protein release [155]. The chaperone complex CSPα/Hsc70/SGT supports SNARE-mediated exosome fusion by preventing SNAP-25 aggregation [156]. Though HSPs are promising biomarkers and therapeutic targets, particularly in cancer, their network complexity necessitates further research to clarify vesicular transport in clinical implications.

The HSP90 dimer on its own has been identified to bind and deform membranes, potentially influencing exosome release [157]. Mutant forms of HSP90 that retain chaperone activity but lack membrane deformation ability showed reduced exosome release, indicating that membrane fusion between multi-vesicular bodies and the plasma membrane is facilitated by HSP90. This process appears to be linked to the ATPase cycle of HSP90 and is promoted by the mentioned previously co-chaperone HOP, which maintains the open conformation necessary for membrane interactions. Despite its substantial role in MVB-to-plasma membrane fusion, HSP90 does not seem to affect other cellular fusion events, suggesting a specific function in exosome release, which is crucial for various biological processes including cancer and neurodegenerative diseases. Eguchi et al. also examined the role of CDC37 in HSP-related vesicle release and EMT in prostate cancer (CRPC) [158]. Vesicles derived from CRPC cells promoted EMT in normal prostate epithelial cells. Some HSP family members and their potential receptor CD91/LRP1 were enriched at high levels in CRPC cell-derived vesicles among over 700 other protein types found by mass spectrometry. Triple siRNA targeting CDC37, HSP90α, and HSP90β was required for efficient reduction in this chaperone trio and to reduce the tumorigenicity of the CRPC cells in vivo, marking a possible target palette for suppressing the secretion of proteins by tumors [159].

The authors of [160] propose that exosomes are part of a HSP90/CHIP/HSP70 network that regulates cell proteostasis. It was demonstrated that the inhibition of CHIP leads to the accumulation of proteasomal substrates and the formation of intracellular protein aggregates. These findings suggest that CHIP inactivation triggers the release of exosomes containing ubiquitinated and oligomerized proteins, serving as a potential mechanism to manage toxic protein accumulation. The study highlights how oxidative stress stimulates exosome release and reflects on the role of CHIP activity in activating HSF1. Although not directly linked to cancer progression yet, the research opens up a notion that CHIP function may drive a shift towards the exosomal clearance of undegraded proteins, and considering impaired oxidative regulation and higher levels of misfolded proteins in neoplasm cells, contribute to overall cancer cell viability.

**Figure 5:**
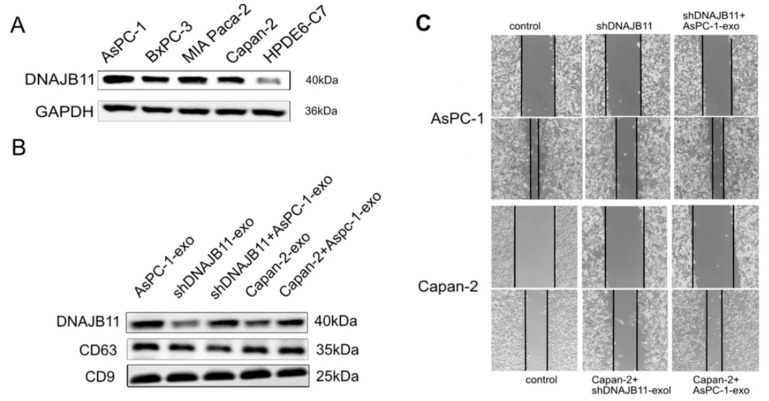
Exosomal DNAJB11 expression is associated with cell proliferation, invasion, and migration in pancreatic cancer cells. (**A**) Western blotting of DNAJB11 expression in pancreatic cancer cell lines and human normal pancreatic cell line (HPDE6C7). GAPDH was used as a control. Three independent experiments were performed. (**B**) PC cells were incubated with exosomes (AsPC-1-exo) and exosome markers CD63 and CD9, and exosomal DNAJB11 levels were measured using Western blotting. Images of the wound healing assay of PC cells with silenced or exosomal DNAJB11 (**C**) [161].

Exosomal DNAJB11, a co-chaperone to HSPA5, significantly influences pancreatic cancer (PC) progression by enhancing HSPA5’s activity and promoting tumor growth (Figure 5) [161]. The study reports that expression levels of DNAJB11 were correlated with advanced disease status and poor survival outcomes in PC patients. Through its interaction with EGFR, DNAJB11 likely facilitates angiogenesis in PC tissues. The study in [162] shows that cells utilize DNAJB6 for exosomal chaperone transport. The proteomic analysis of vesicles from different cell lines revealed that DNAJB6 overexpression enhances the loading of chaperone proteins, which could improve proteostasis and alleviate associated stress. Conversely, a mutant form of this protein showed reduced chaperone loading and the downregulation of unrelated proteins. Although this research was conducted with neurodegenerative diseases in mind, the release of exosomes containing chaperones during cellular stress may be of interest in researching cancer progression and early noninvasive diagnostics.

Protein IQGAP1, a part of the HSPA1A interactome, is known for its contribution in cell junction and polarity control, being a RAS-related GTPase modulator. Recently, a mechanistic connection between IQGAP1 and exosome biogenesis was proposed through a gasdermin D link [163] in epithelial cells. If the same observations follow for cancer cases, a connection between HSPA1A, IQGAP1, and Tsg101 may reveal crucial information on tumor exosome formation and cargo sorting. The study in [164] actually identifies the Cdc42-IQGAP1 signaling pathway as a regulator of tumor microvesicle biogenesis, with Cdc42 being activated by upstream signals like EGF. The investigation highlights the complexity of vesicle formation involving various signaling pathways, including the roles of Arf6 and RhoA, showing that multiple pathways function together to control microvesicle release. The inhibition of clathrin-mediated endocytosis was found to enhance this effect release under EGF stimulation, while Cdc42 is a critical node in these regulatory processes. Overall, it suggests that manipulating tumor vesicle shedding through HSP90-Cdc42 could have implications for cancer therapy.

TIMP2 has been identified as a partner of extracellular HSP90, playing a significant role in cancer-related stress responses [165]. It is upregulated during stress, increasing the levels of extracellular ATP and affecting matrix degradation and tumor cell invasiveness. TIMP2 inhibits the ATPase activity of HSP90, potentially preventing conformational changes that would activate it, while competing with AHA1. The interplay between TIMP2 and Aha1 regulates the extracellular MMP2:HSP90 complex, with TIMP2 serving to disrupt MMP2 activity, thus influencing cancer progression. This dynamic suggests that extracellular co-chaperones TIMP2 and Aha1 have opposing effects on client stability and activity. Both these proteins were found to be secreted in vesicles, introducing a new point of interest in investigating matrix dynamics in tumorigenesis.

Recent studies also highlight the complex involvement of HSPA1A-containing extracellular vesicles in the immune response to tumors. Vesicles from heat-treated tumor cells exhibit strong antitumor activity, eliciting an immune response. HSPA1A from resistant cancer cells stimulates an NK-cell antitumor response. Studies indicated that HSPA1A is released from melanoma cells in both a soluble form and within vesicles, effectively sensitizing these tumor cells to NK cell cytolytic activity [166]. In vivo experiments with melanoma and colon carcinoma models demonstrated that HSPA1A-containing exosomes reduce tumor growth and enhance survival by activating cytotoxic CD8+ T-cell responses and altering cytokine levels. The authors suggest that these effects are due to the influence of HSP on the microenvironment rather than the tumor cells directly, indicating their potential therapeutic role in modulating antitumor immunity.

Overall, we can see a growing number of experimentally backed associations between vesicle traffic in tumor progressions and chaperome members. Though complex and varying between physiological cell states, co-chaperones show involvement in extracellular vesicle biogenesis and cargo control, supported by the ability of HSP90 to influence membrane geometry. At the same time, extracellular forms of chaperones and co-chaperones contribute to tumor microenvironment dynamism, possibly integrating with immune response and matrix remodeling systems.

## 6. Conclusions

The unique central role of HSPs in cell proteostasis has encouraged these chaperones and their related systems to be widely researched in different contexts: cell stress and adaptivity, their involvement in cell death, and in the case of cancers, cell viability, and motility. Currently we understand several HSP systems (HSPA, HSPC) as interfacing machineries regulated by key co-chaperones. The conformational change and co-chaperone binding and release support necessary substrate recruitment and folding, followed by localization. However, this process is not the extent of HSP and co-chaperone functionality. With an expanding experimental base, we now have insights into HSP interactome involvement in different crucial cellular events.

Several co-chaperones (e.g., ARHGEF, DNAJ, Aha, HOP) are involved in regulating tumor cell motility by modulating the GTPases responsible for cytoskeleton reorganization. Experiments have linked the enhanced expression of these proteins within the HSP network with key signaling pathways (RHOA, MEK, MAPK) and illustrated their activity with cell motility assays. Some proteins with more distant relations to the chaperone cycle (CK2, FAF1, RAF1) were still found to be contributing factors in HSP-related signaling and proteostasis processes, at the same time exhibiting involvement in tumor progression. As these interactome members were also shown to have links to EMT in various cancer cell lines, a network approach for investigating the pathogenic characteristics of chaperone proteins may help form a scientific basis. Additionally, as extracellular vesicle research grows in availability and interest, we will see a contribution of HSP and co-chaperones to extracellular experiments. HSP90 itself plays a big role in vesicle genesis and shedding, supported by Cdc42 and 37 co-chaperones. Organizing HSP90-to-HSP70 links into secreted forms, HOP, IQGAP1, and CHIP present potential as a theranostic palette for targeting. Although many of the described phenomena are not mechanistic, being based on correlation and co-occurrence, they still build upon necessary information to help researchers understand crucial influence points for tumor progression. Based on the available approaches, we constructed a list of employable assays in HSP interactome research in the cancer context (Table 1).

**Figure 6:**
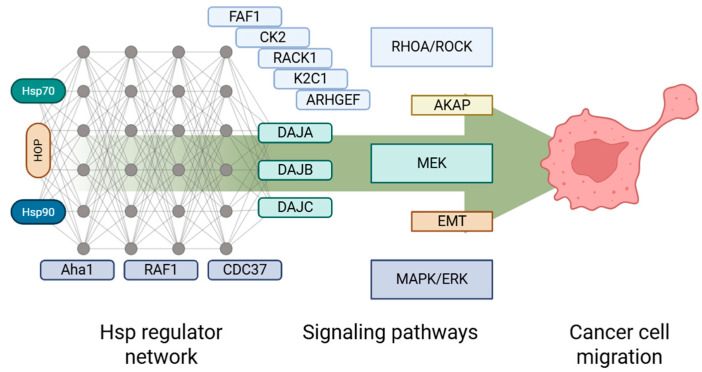
Recent links between co-chaperones and migration-enabling signaling pathways. Conceptual schematic; no quantitative data shown.

Recent studies are able to delve into deeper details concerning the HSP interactome and its dynamics. Proteome- and cell-level experiments show that the same proteins, known as HSP co-chaperones, are embedded in different signaling pathways, most of which are responsible for tumor development and metastasis (Table 2). A schematic of co-chaperone–signaling interplay is presented in Figure 6. With EMT and rapid cytoskeleton reorganization being a staple cell event in the cancer context, developing a palette of crucial hubs within the HSP interactome as a target for multi-faceted inhibitors or SiRNA therapy may be a suitable strategy for impairing tumor progression.

## Figures and Tables

**Table 1:** HSP network analysis approaches.

Research Task	Experimental Approach
Gene expression evaluation and co-occurrence evaluation	Flow cytometry
	Transcriptomics
	Western blot assays
	Fluorescent staining
Gene expression control	siRNA
	Heat shock
	Oxidative stress
	Exogenous HSP and co-chaperones (vesicle or soluble)
	Low electric treatment
Network profiling	Mass spectrometry
	Western blot assay
	Inhibitory assays
Cell motility assessment	Single cell tracking
	Wound healing assay
	Inhibitory assays
	Transwell assay

**Table 2:** HSP co-chaperones’ involvement with tumor progression nodes.

HSP Node	Co-Chaperone/Interactor	Affected Node	Evidence Level	References
HSPA1A	RACK1	AKT/P53	In vivo	[127,128,167]
HSPA1A	DNAJA1,3	P53, MMPs	In vivo	[133,134,136,168,169]
HSPA1A	DNAJB1,4,6	P53, MMPs	In vitro/In vivo (DNAJB6)	[134,162,170,171]
HSPA1A	DNAJB11 (exosomal)	MAPK pathway	In vivo	[161]
HSPA1A	BAG2	P53/PKA	In vivo	[137,172,173]
HSP90	AHA1	AKT/MMPs	In vitro	[140,165]
HSP90	CDC37	CDK6/RAF1	In vitro	[149,158,159]
HSP90/70	HOP	RAF1/MMPs	In vivo	[144,145,146,148]
HSP90/70	CHIP	HSF	In vitro	[80,160,173]

## Data Availability

No new data were created or analyzed in this study. All figures were reused under CC BY 4.0 from open-access journals.

## Abbreviations

The following abbreviations are used in this manuscript:

HSPs	Heat Shock Proteins
ECM	Extracellular Matrix
HUGO	Human Genome Organization
DAMP	Damage-Associated Molecular Pattern
NBD	Nucleotide-Binding Domain
SBD	Substrate-Binding Domain
MD	Middle Domain
CTD	C-terminal Domain
TPR	Tetratricopeptide Repeat
NEFs	Nucleotide Exchange Factors
GRP	Glucose-regulated Protein
ER	Endoplasmic Reticulum
HOP	Hsp-Organizing Protein
STIP	Stress-induced Protein
JDPs	J-Domain Proteins
EMT	Epithelial–mesenchymal transition
LET	Low Electric Treatment
cSCC	Squamous cell Carcinoma
GR	Glucocorticoid Receptor
PPI	Peptidyl-prolyl isomerase
GEF	Guanine nucleotide Exchange Factor
GAP	GTPase-activating Protein
FKBP	FK506-binding Protein
GC	Gastric Cancer
NSCLC	Non-small cell Lung Cancer
CRPC	Castration-resistant prostate cancer
EGF	Epithelial growth factor
HSF	Heat shock factor
GDI	Guanine nucleotide Dissociation Inhibitor
ESCRT	Endosomal Sorting Complex Required for Transport
MMP	Matrix metalloproteinase
